# Complete Genome Sequence of Rhodobacter sphaeroides Strain HJ, a Purple Nonsulfur Bacterium with the Ability To Produce High Levels of Hydrogen from Acetate

**DOI:** 10.1128/MRA.00652-19

**Published:** 2019-09-12

**Authors:** Jyumpei Kobayashi, Akihiko Kondo

**Affiliations:** a Graduate School of Science, Technology and Innovation, Kobe University, Kobe, Japan; b Department of Chemical Science and Engineering, Graduate School of Engineering, Kobe University, Kobe, Japan; c RIKEN Center for Sustainable Resource Science, Yokohama, Japan; University of Southern California

## Abstract

Previously, Rhodobacter sphaeroides strain HJ was isolated to obtain a purple nonsulfur bacterium with the ability to produce high levels of hydrogen from acetate. However, the genome of this strain has not been previously sequenced. Therefore, the complete genome sequence of R. sphaeroides strain HJ is presented in this report.

## ANNOUNCEMENT

Rhodobacter sphaeroides is a purple nonsulfur (PNS) bacterium, which has many unique characteristics useful in bioproduction. Among the various products of PNS bacteria, hydrogen has recently attracted attention as a clean alternative to fossil fuels. In previous study, we screened PNS bacteria with high hydrogen production ability from lactate and acetate and obtained R. sphaeroides strain HJ from ponds in Tokyo, Japan (1). Although R. sphaeroides HJ was recently used as a host bacterium for polyhydroxyalkanoate production instead of hydrogen (2), its genome has not yet been sequenced. To make research on R. sphaeroides HJ easier, we present the genome sequence of this PNS bacterium that has the ability to produce high levels of hydrogen.

Genomic DNA of R. sphaeroides strain HJ was extracted from cells grown in ammonium sulfate, succinate, and yeast extract (ASY) liquid medium (1) using NucleoBond AXG 20 columns (TaKaRa Bio, Otsu, Japan) and NucleoBond buffer set III (TaKaRa), following the manufacturer’s instructions. The DNA quality and quantity were determined using a NanoDrop instrument (Thermo Fisher Scientific, CA, USA), a Quant-iT double-stranded DNA (dsDNA) broad-range (BR) assay kit (Thermo Fisher Scientific), and agarose gel electrophoresis. The extracted DNA was fragmented to about 20 kb using a g-TUBE (Covaris, MA, USA), and these fragments were used for preparing a library with the SMRTbell template prep kit 1.0 (Pacific Biosciences, CA, USA). The size of the library was selected using BluePippin (Sage Science, MA, USA), and the quality of the library was checked using the Agilent 4200 TapeStation system (Agilent Technologies, CA, USA). The library was bound to polymerase using the DNA/polymerase binding kit P6 v2 (Pacific Biosciences). The DNA polymerase/template complex was further bound to magnetic beads using MagBead kit v2 (Pacific Biosciences), loaded onto a single-molecule real-time (SMRT) cell (Pacific Biosciences), and then sequenced using a PacBio RS II instrument (Pacific Biosciences). The obtained data were assembled *de novo* using SMRT Analysis attached to a PacBio RS II instrument according to the Hierarchical Genome Assembly Process (HGAP) workflow, including preassembly and consensus polishing. In HGAP data processing (3), PreAssembler Filter v1 (minimum subread length, 500 bp; minimum polymerase read quality, 0.80; minimum polymerase read length, 100 bp), PreAssembler v2 (compute minimum seed length, true; number of seed read chunks, 6; alignment candidates per chunk, 10; total alignment candidates, 24; minimum coverage for correction, 6), AssembleUnitig v1 (genome size, 4,600,000 bp; target coverage, 25; overlapper error rate, 0.06; overlapper minimum length, 40 bp; overlapper k-mer, 14), BLASR v1 (maximum divergence, 30; minimum anchor size, 12), and AssemblyPolishing v1 (use only unambiguously mapped reads, true) were used for filtering, assembling, mapping, and consensus polishing, respectively. Other parameters were kept at the defaults. After the whole genome was assembled, gene functions were annotated via the NCBI Prokaryotic Genome Annotation Pipeline (https://www.ncbi.nlm.nih.gov/genome/annotation_prok/).

The raw data generated a total of 1,490,875,792 bases and 131,073 reads, with an *N*_50_ length of 19,344 bp by filtering. The assembled genome of R. sphaeroides strain HJ comprises two chromosomes and a single plasmid in three contigs, and the overlapping ends of these chromosomes and plasmid were manually removed to finish circularization, resulting in two circular chromosomes designated chromosome 1 (3,263,112 bp with about 234-fold coverage) and chromosome 2 (1,186,739 bp with about 227-fold coverage) and a single plasmid (96,568 bp with about 97-fold coverage). These chromosomes and plasmid were determined by comparison with other replicons of R. sphaeroides strains deposited in the NCBI database. The G+C contents of chromosomes 1 and 2 and the plasmid were 69.2%, 68.8%, and 71.0%, respectively. The HJ strain carries 55 tRNAs, 1 transfer-messenger RNA (tmRNA), 12 rRNAs, and 4,353 protein-coding genes. These genetic features are common among PNS bacteria, and R. sphaeroides strain HJ shares many characteristics with other PNS bacteria (4–6). It is unclear why R. sphaeroides strain HJ produces large amounts of hydrogen from acetate, but these genome sequences provide important information for future research.

### Data availability.

The results of this whole-genome project have been deposited in DDBJ/ENA/GenBank under the accession numbers CP036419, CP036420, and CP036421 and under BioProject accession number PRJNA523284. The accession number for the raw reads is SRS4788417. The versions described here are the first versions.

## References

[1] Kobayashi J, Yoshimune K, Komoriya T, Kohno H 2011 Efficient hydrogen production from acetate through isolated *Rhodobacter sphaeroides*. J Biosci Bioeng 112:602–605. doi:10.1016/j.jbiosc.2011.08.008. 21903465

[2] Kobayashi J, Kondo A 2019 Disruption of poly (3-hydroxyalkanoate) depolymerase gene and overexpression of three poly (3-hydroxybutyrate) biosynthetic genes improve poly (3-hydroxybutyrate) production from nitrogen rich medium by *Rhodobacter sphaeroides*. Microb Cell Fact 18:40. doi:10.1186/s12934-019-1088-y. 30808422 PMC6390342

[3] Chin CS, Alexander DH, Marks P, Klammer AA, Drake J, Heiner C, Clum A, Copeland A, Huddleston J, Eichler EE, Turner SW, Korlach J 2013 Nonhybrid, finished microbial genome assemblies from long-read SMRT sequencing data. Nat Methods 10:563–569. doi:10.1038/nmeth.2474. 23644548

[4] Guzman MS, Bose A 2019 Draft genome sequences of four *Rhodobacter sphaeroides* strains isolated from a marine ecosystem. Microbiol Resour Announc 17:e01648-18. doi:10.1128/MRA.01648-18. PMC6346176 30687844

[5] Ding H, Moksa MM, Hirst M, Beatty JT 2019 Draft genome sequences of six *Rhodobacter capsulatus* strains, YW1, YW2, B6, Y262, R121, and DE442. Microbiol Resour Announc 13:e00050-14. doi:10.1128/genomeA.00050-14. PMC3924369 24526637

[6] Anton BP, Roberts RJ, Fomenkov A, Humbert A, Stoian N, Zeilstra-Ryalls J 2018 Complete genome sequences of two *Rhodobacter* strains. Microbiol Resour Announc 27:e01162-18. doi:10.1128/MRA.01162-18. PMC6256690 30533667

